# Mislocalization of p27 to the cytoplasm of breast cancer cells confers resistance to anti-HER2 targeted therapy

**DOI:** 10.18632/oncotarget.2871

**Published:** 2015-01-03

**Authors:** Hui Zhao, Claire M. Faltermeier, Lori Mendelsohn, Peggy L. Porter, Bruce E. Clurman, James M. Roberts

**Affiliations:** ^1^ Basic Sciences Division, Fred Hutchinson Cancer Research Center, Seattle, Washington, USA; ^2^ Biology Department, Whitman College, Walla Walla, Washington, USA; ^3^ Human Biology Division, Fred Hutchinson Cancer Research Center, Seattle, Washington, USA; ^4^ Public Health Sciences Division, Fred Hutchinson Cancer Research Center, Seattle, Washington, USA; ^5^ Clinical Research Division, Fred Hutchinson Cancer Research Center, Seattle, Washington, USA

**Keywords:** p27^Kip1^ (p27), cell proliferation, cytoplasmic localization, Her2+ breast cancer cells, lapatinib, drug sensitivity

## Abstract

As a cell cycle inhibitor and tumor suppressor, p27 is frequently misregulated in human cancers. Increased degradation is the most common mechanism of misregulation, however in some cancers, p27 is mislocalized from its cell cycle inhibitory location in the nucleus, to the cytoplasm. In normal cells cytoplasmic p27 has functions that are distinct from its cell cycle-regulatory nuclear functions. Therefore, an important question is whether localization of p27 to the cytoplasm in tumor cells is primarily a mechanism for cancelling its inhibitory effect on cell proliferation, or whether cytoplasmic p27 has more direct oncogenic actions. To study p27 mislocalization in human cancers we screened a panel of common breast cancer cell lines. We observed that p27 accumulated in the cytoplasm exclusively in cell lines that are Her2+. To address the significance of p27 mislocalization in Her2+ breast cancer cells we interrogated the cellular response to the dual-Her2/EGFR kinase inhibitor, lapatinib. Knockdown of p27 using shRNA sensitized Her2+ cells to lapatinib-induced apoptosis. Moreover, expression of a constitutively cytoplasmic form of p27 (p27ΔNLS) reversed the lapatinib-induced apoptosis, suggesting that cytoplasmic p27 contributed to lapatinib resistance in Her2+ breast cancer cells by suppressing apoptosis. Our results suggest that p27 localization may be useful as a predictive biomarker of therapeutic response in patients with Her2+ breast cancers.

## INTRODUCTION

p27^Kip1^ (hereafter p27) negatively regulates the G1 to S phase progression by binding to and inhibiting cyclin dependent kinases (CDKs) [1]. p27 knock out mice have multiorgan hyperplasia and spontaneously develop pituitary tumors, supporting the tumor suppressor role of p27 [2–4]. Unlike other cell cycle inhibitors such as p16 and p21, which are frequently mutated or deleted in human cancers, genetic alterations of p27 are rare. Rather, p27 is misregulated in cancers by transcriptional and post-transcriptional mechanisms [5, 6]. Likewise, low levels of p27 correlates with poor prognosis and survival in many types of cancers.

Surprisingly, p27-haploinsufficient mice are more sensitive to ErbB2/Neu induced transformation compared to p27 null mice [7]. Similarly, in mice lacking the homeobox gene Nkx3.1 and the tumor suppressor PTEN, prostate carcinogenesis was enhanced when one allele of p27 was lost, while cancer progression was inhibited when both alleles of p27 were deleted [8]. These observations suggest that while p27 is a tumor suppressor, partial loss of p27 function is more oncogenic than complete loss, although the mechanisms for this remain unclear.

The finding that p27 has functions in addition to its role as a cell cycle inhibitor and tumor suppressor has important implications for understanding p27 misregulation in cancer. Mice expressing a form of p27 that is unable to bind or inhibit cyclin-CDK complexes, known as p27CK-, develop hyperplastic lesions and tumors in multiple organs. Moreover, the pituitary tumors in these mice are more aggressive compared to p27 null mice, suggesting p27 may have oncogenic functions [9]. How p27 changes it role from a tumor suppressor to a tumor promoter is poorly understood, but this may result when p27 is mislocalized to the cytoplasm. Indeed, p27CK- was detected in the cytoplasm in urethane induced lung tumors, and cytoplasmic localization of p27 correlated with K-Ras induced cell transformation [10, 11]. In MCF7 breast cancer cells, when a mutant form of p27 which exclusively localized to the cytoplasm was overexpressed, cell motility and survival increased [12]. Likewise, knock down of p27 in a glioma cell line in which p27 is localized predominantly to the cytoplasm decreased tumorigenicity [12]. Consistently, cytoplasmic expression of p27 induced melanoma motility and metastases [13], and nuclear sequestration of p27 by PI-3K inhibition reduced tumorigenesis in a mouse lung cancer model [14]. Cytoplasmic p27 contributes to cell migration by interacting with the GTPase RhoA and the microtubule destabilizing protein Stathmin and this may, in part, promote tumor progression and metastasis [15–17].

Cytoplasmic p27 has been detected in many human cancers, including melanoma, ovarian carcinoma, renal cell carcinoma, osteosarcoma, acute myelogenous leukemia, and breast cancer. Mislocalization of p27 from the nucleus to the cytoplasm is generally associated with poor prognosis and survival, high tumor grade and metastasis [13, 18–22], suggesting a potential application of cytoplasmic p27 as a clinical prognostic marker. However, the mechanisms by which cytoplasmic p27 is oncogenic, and may impact responses to therapeutics, remain incompletely understood.

Multiple mechanisms misregulate p27 in tumors. Increased expression of Skp2, the predominant ubiquitin ligase that targets p27 for degradation, has been correlated with decreased p27 levels and poor prognosis in young women with breast cancer and in patients with Her2− breast tumors [23, 24]. Also, cytoplasmic p27 in some primary breast tumors has been correlated with activation of the serine/threonine kinase, AKT. Since p27 is a substrate for AKT, it has been proposed that p27 is sequestered in the cytoplasm through phosphorylation on T157 [20, 25, 26].

Amplification of Her2 occurs in 15–20% of breast tumors and correlates with poor prognosis [27, 28]. Treatment of Her2+ breast cancer cells with the Her2−specific antibody trastuzumab or the dual-Her2/EGFR inhibitor Lapatinib results in cell cycle arrest, possibly due to increased p27 protein levels [29]. However, the role of cytoplasmic p27 in breast cancer and targeted therapy hasn't been fully assessed. Here we show that p27 becomes mislocalized to the cytoplasm specifically in Her2+ breast cancer cells released into cell cycle from mitogen deprivation induced arrest. As expected Lapatinib treatment blocked Her2+ cell proliferation, and also prevented p27 cytoplasmic mislocalization. Moreover p27 silencing by shRNA decreased lapatinib-induced cell cycle arrest, indicating that nuclear p27 contributes to this arrest. All of these results were consistent with p27's canonical tumor suppressor function as a CDK-inhibitor. However, we also found that cytoplasmic p27 suppressed lapatinib-induced apoptosis, and that the net effect of decreasing p27 was to sensitize Her2+ cancer cells to lapatinib. This indicated, for the first time, that the tumorigenic effect of cytoplasmic p27 might be mediated by a suppression of apoptosis, which we suggest may cause increased resistance to anti-tumor therapies that induce cancer cell death.

## RESULTS

### P27 is mislocalized to the cytoplasm in Her2+ breast cancer cells

**Figure 1:**
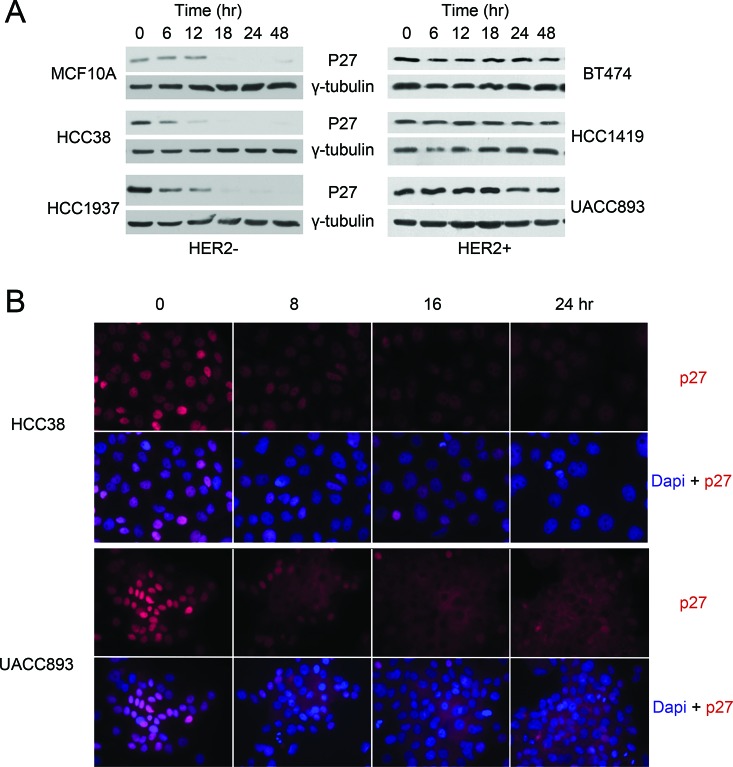
P27 mis-localizes to the cytoplasm in HER2+ breast cancer cells Representative western blot **(A)** and immunofluorescence staining **(B)** of p27 in Her2− and HER2+ cells harvested at the indicated time points after release from serum starvation.

To investigate p27 regulation in human breast cancer, we compared p27 protein levels in quiescent (serum starved) and proliferating cells in a panel of Her2− and Her2+ breast cancer cell lines (Fig. 1A). Cells were synchronized by serum starvation and released into the cell cycle upon mitogen stimulation. As seen in normal cells, p27 protein abundance decreased when the immortalized, but non-tumorigenic breast cell line MCF10A was stimulated with mitogens to enter the cell cycle. A similar pattern was seen in breast cancer cell lines representing subtypes other than Her2+ breast cancers (Fig.1A and data not shown). Unexpectedly, p27 remained abnormally high in Her2+ cell lines, even after 48 hrs of serum stimulation. Consistent with the western blots, immunofluorescence staining showed that p27 steadily declined when Her2− cells begin proliferating (Fig. 1B, HCC38, Supplemental Fig.1, HCC1395 and data not shown). In contrast, whereas nuclear p27 abundance decreased in Her2+ cells, p27 accumulated in the cytoplasm upon serum stimulation (Fig. 1B, UACC893, Supplemental Fig.1, HCC1419 and data not shown). In addition, cells located in the middle of the cell cluster, an area where cell proliferation is inhibited by contact inhibition, exhibited the largest amount of cytoplasmic p27. These data suggested that p27 was regulated differently during the cell cycle depending on Her2+ status. That is, p27 is degraded in the nucleus as Her2− breast cancer cells and MCF10A cells progress through the cell cycle, but is instead mislocalized to cytoplasm in Her2+ cells.

### Lapatinib induces p27 nuclear re-distribution and cell cycle arrest in Her2+ breast cancer cells

**Figure 2:**
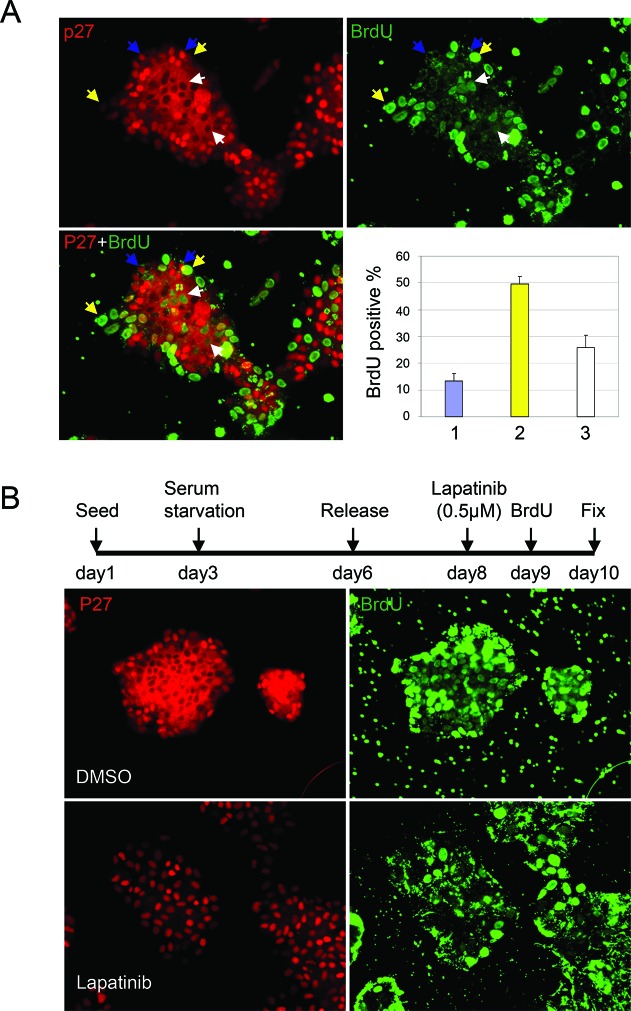
Lapatinib induces p27 re-distribution and cell cycle arrest in HER2+ breast cancer cells **(A)** Co-staining of p27 and BrdU in UACC893 cells which were pulse labeled with BrdU for 5 hr following 48 hrs after release from serum starvation. Cells were divided into three categories: 1) cells with high nuclear p27 (indicated by blue arrows), 2) cells with low p27 in both nuclear and cytoplasm (indicated by yellow arrow) and 3) cells with p27 exclusively in the cytoplasm (indicated by white arrow). BrdU positive cells from at least 10 cell clusters were quantified. The average percentage of BrdU positive cells/cluster in each category were graphed (error bars-STDEV). **(B)** Co-staining of p27 and BrdU in UACC893 cells treated as depicted. Cells were pulse labeled with BrdU for 13 hr.

We investigated the relationship between p27 localization and cell proliferation by labeling cells with BrdU for 5 hr and co-immunostaining the cells with p27 and BrdU antibodies (Fig. 2A). Cells were classed in three categories: those with p27 exclusively in the cytoplasm (indicated by white arrow), with high nuclear p27, (indicated by blue arrow), and with low p27 in both the nucleus and cytoplasm (indicated by yellow arrow). Consistent with its cell cycle inhibitory role in the nucleus, BrdU was rarely detected in cells with high nuclear p27 (blue arrow). Quantification of 50 cell clusters (80–150 cells per cluster) indicated that 12% of the cells with high nuclear p27 were BrdU positive, whereas 50% of the cells with low nuclear (yellow arrow) had incorporated BrdU. Cells with p27 exclusively localized to the cytoplasm (white arrow) also hyper-proliferated (25% BrdU positive). Cytoplasmic p27 thus correlated with significantly increased cell proliferation compared with nuclear p27.

Her2 inhibition with therapeutics such as trastuzumab or lapatinib leads to an increase in p27 protein levels, which is speculated to be important for these reagents' anti-proliferative effects [29]. We thus investigated the response of Her2+ cells to lapatinib treatment by co-immunostaining of p27 and BrdU. We found that cell proliferation stopped upon Lapatinib treatment, as measured by BrdU incorporation (Fig. 2B). Even after a 13 hr pulse labeling, very few cells were BrdU positive. Importantly, lapatinib treatment also caused these cells, which exhibit p27 cytoplasmic mislocalization, to express nuclear rather than cytoplasmic p27.

**Figure 3:**
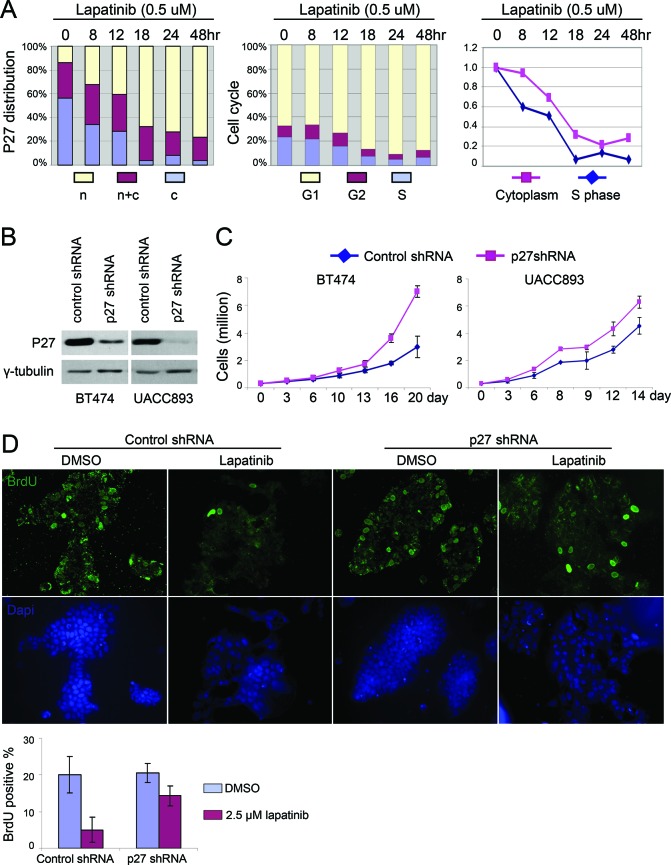
Lapatinib induced nuclear accumulation of p27 is required for its anti-proliferative effect **(A)** UACC893 cells were released from serum starvation for 48 hr. Lapatinib was added and samples from indicated time points were collected for cell cycle analysis and p27 immunostaining. For subcellular distribution of p27, 50 cell clusters were quantified to determine the ratio of the number of cells with predominantly nuclear p27 to cells with exclusively cytoplasmic p27 in each cluster. N: ratio > 1; N + C: ratio = 1; C: ratio < 1. Right panel shows the aligned time course trend for cells in S phase and cells with p27 exclusively in the cytoplasm. **(B)** Western blot showing knock down of p27 with p27 shRNA in BT474 and UACC893 cells. **(C)** Proliferation of control and p27-knockdown cells. Experiments were repeated for three times, and averaged (error bars-STDEV). **(D)** BrdU pulse label and immunostaining. UACC893 Cells were treated as depicted in Figure 2B, except that BrdU was added 7 hr before fixation. BrdU positive cells were counted from five cell clusters (each cluster contains 100–700 cells). The average numbers of positive cells are graphed (error bars-STDEV)

We next determined if nuclear p27 accumulation played a causal role in lapatinib-mediated cell cycle arrest, or was simply a secondary effect of cell cycle arrest. First, we examined p27 expression during cell cycle re-entry to address whether lapatinib induced nuclear p27 before or after cell cycle arrest. At the indicated time points after lapatinib treatment, cells were fixed to evaluate p27 localization by immunostaining or BrdU pulse labeled (2 hrs) to evaluate their cell cycle distribution by flow cytometry (Fig. 3A). Whereas the percentage of cells in S phase didn't significantly drop until 12 hrs after treatment with lapatinib, nuclear p27 accumulation began as early as 8 hrs after treatment, at which point the number of cell clusters exhibited nuclear p27 in most cells increased from 14% to 32%. Thus p27 nuclear accumulation preceded cell cycle arrest, supporting the hypothesis that p27 was a cause, rather than a consequence, of lapatinib's anti-proliferative effect.

To directly determine the requirement for p27 expression in lapatinib-induced cell cycle arrest, we knocked down p27 with shRNA in UACC893 and BT474 cells (Fig. 3B). As expected, decreased levels of p27 led to an increased rate of proliferation, as evaluated by cell number (Fig. 3C). We then synchronized cells by serum starvation/restimulation and examined the effect of p27 shRNA on the ability of lapatinib to arrest the cell cycle. Unlike the control cells, which underwent cell cycle arrest upon lapatinib treatment, a much greater percentage of cells expressing p27 shRNA entered S-phase, as shown by incorporation of BrdU (Fig. 3D). Induced nuclear accumulation of p27 thus appeared essential for laptinib's anti-proliferative effect.

### ShRNA inhibition of p27 sensitizes Her2+ cells to lapatinib-induced apoptosis

**Figure 4:**
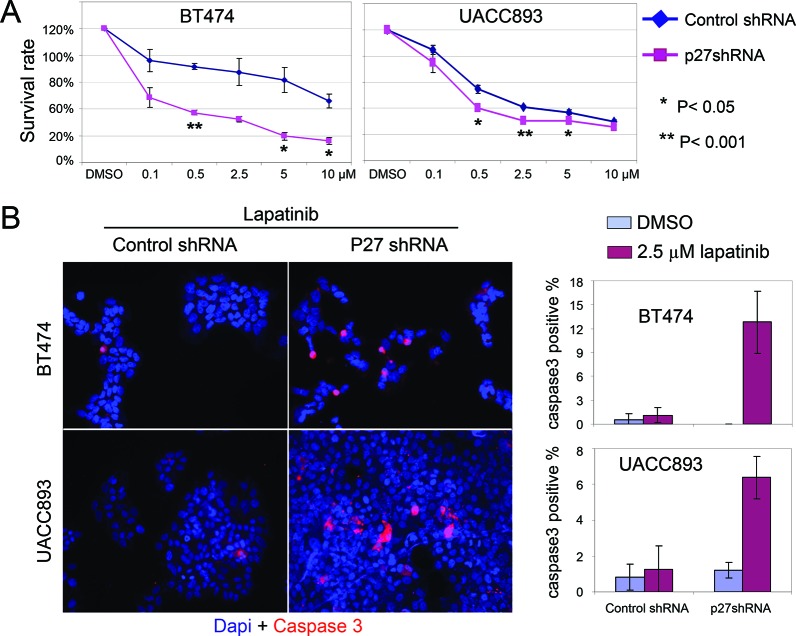
Knock down of p27 sensitizes HER2+ cells to lapatinib-induced apoptosis **(A)** Lapatinib sensitivity assay. Cells released from serum starvation were treated with the lapatinib at the indicated concentrations for 72 hr. Viable cells were identified with trypan blue staining, and normalized to control cells (treated with DMSO). The graph depicts the average of three independent experiments (error bars-SEM). **(B)** UACC893 or BT474 Cells were treated as (A), except treated with DMSO or lapatinib (2.5 μM) for 48 hr. Caspase3 positive cells were counted from at least 10 cell clusters (for UACC893 cells, each cluster contains 100–700 cells) or random fields (for BT474, each fields contains 60–300 cells). The averages numbers of positive cells were graphed (error bars-STDEV).

Our observations raised the question whether cytoplasmic p27 in Her2+ tumor cells had potentially oncogenic consequences beyond simply reducing nuclear (anti-proliferative) p27. To address this, we measured the effect of lapatinib on the viability of UACC893 and BT474 cells after p27 knockdown. Remarkably, we found that p27 knockdown rendered Her2+ cells hypersensitive to lapatinib treatment, as measured by the number of viable cells 72 hrs after drug treatment (Fig. 4A). As shown in Fig. 4B, p27 knockdown caused increased apoptosis after lapatinib treatment in both UACC893 and BT474 cells, as shown by cleaved-caspase3 (hereafter caspase3) immunostaining. This increase in apoptosis counter-acted the increased ability of p27-knockdown cells to enter S phase, the net effect being increased drug sensitivity.

**Figure 5:**
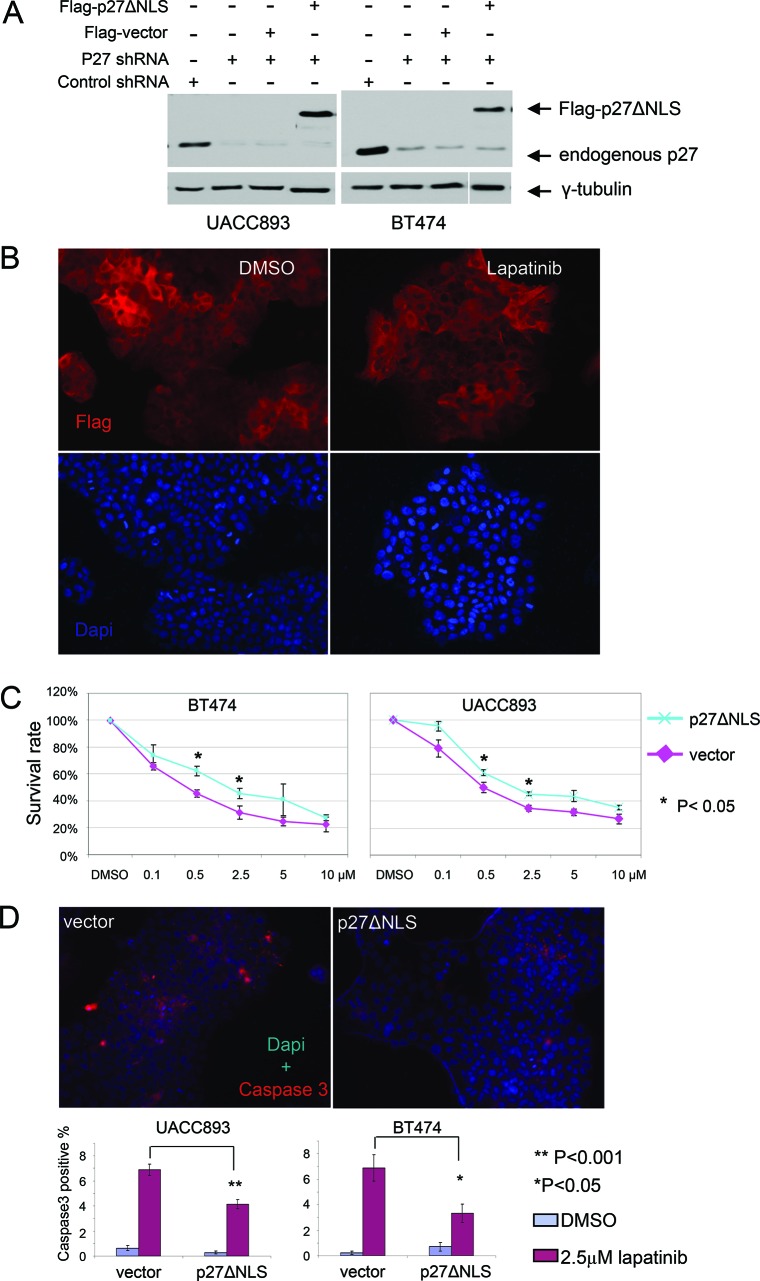
Re-expression of cytoplasmic p27 de-sensitizes HER2+ cells to lapatinib **(A)** Western blot showing stable expression of Flag-p27ΔNLS in p27 shRNA infected BT474 and UACC893 cells. **(B)** Flag-p27ΔNLS remains in the cytoplasm during lapatinib treatment. UACC893 cells expressing p27 shRNA and Flag-p27ΔNLS were immunostained with anti-Flag antibody. Cells were treated with DMSO or 0.5 μM of lapatinib for 48 hr as in Figure 4B. **(C)** Lapatinib sensitivity assay. Experiment was performed as in Figure 4A. **(D)** Caspase3 immunostaining of UACC893 and BT474 cells treated with DMSO or lapatinib (2.5 μM, 48 hr). Caspase3 positive cells were counted from 10 cell clusters (for UACC893 cells, each cluster contains 100–500 cells) or at least 10 random fields (for BT474 cells, each field contains 40–200 cells). The averages were graphed (error bars- SEM). The representative images show lapatinib treated cells.

These results suggested that cytoplasmic p27 was not simply null with respect to function, but might promote tumorigenesis by suppressing cell death. Alternatively, increased cell death in p27 knockdown cells may be an indirect effect caused by inappropriate cell cycling after exposure to lapatinib. To distinguish between these two hypotheses, we expressed a constitutively cytoplasmic p27 mutant (p27ΔNLS) in UACC893 and BT474 cells, in which endogenous p27 expression was silenced by shRNA that did not target the ectopic p27 expression. We confirmed that p27ΔNLS did not enter the nucleus, and was unable to inhibit cell proliferation [12]. p27ΔNLS was expressed at levels comparable to endogenous p27 (Fig. 5A) and localized predominantly in the cytoplasm, even after lapatinib treatment (Fig. 5B). Importantly the replacement of endogenous p27 with P27ΔNLS reversed lapatinib sensitivity in BT474 and UACC893 cells (Fig. 5C) and reduced lapaitinib-induced apoptosis in UACC893 and BT474 cells in which endogenous p27 had been knocked-down (Fig. 5D). Thus cytoplasmic p27 expression directly suppressed lapatanib-induced apoptosis in Her2+ breast cancer cell lines.

## DISCUSSION

Cytoplasmic p27 is associated with poor prognosis in multiple human cancers including breast cancer, but its mechanisms of mislocalization and role in therapeutic responses are poorly understood. In this study, we examined Her2+ breast cancer cells, in which we found that p27 was mislocalized in the cytoplasm, and this finding is supported by Andre et al., who reported that a low p27 nuclear/cytoplasmic ratio correlated with Her2 expression in human breast cancer [30]. In addition to its role as a cell cycle inhibitor, p27 also regulates apoptosis. We previously found that p27 depletion caused increased apoptosis in cells deprived of growth factors and that CDK2 activity was consistently enhanced in these cases, suggesting that the CDK2 inhibition by p27 is instrumental in blocking apoptosis [31, 32]. Here we show that treatment of Her2+ breast cancer cell lines with lapatinib blocks cell proliferation, and that this is associated with increased nuclear p27 abundance in conjunction with decreased cytoplasmic p27. These results suggested that nuclear p27 relocalization was required for lapatinib induced cell cycle arrest, which we confirmed by using p27shRNA to prevent p27 nuclear accumulation. Although p27 knockdown caused lapatinib-treated Her2+ cells to continue to proliferate, this was also associated with increased apoptosis, consistent with the role of enhanced CDK2 activity in promoting apoptosis. The combined impact of these seemingly opposite consequences of p27 knockdown (increased proliferation and increased apoptosis) was to increase the sensitivity of Her2+ cells to lapatinib treatment.

Because Her2+ breast cancer cells exhibit p27 mislocalization rather than reduced p27 abundance, we determined the role of p27 cytoplasmic mislocalization in apoptotic responses by expressing a constitutively cytoplasmic p27 mutant, which actively suppressed lapatinib-induced apoptosis. These data suggested that in addition to preventing its role as a CDK inhibitor in the nucleus, cytoplasmic p27 mislocalization had another role in determining lapatinib responses, by actively suppressing apoptosis. However, since cytoplasmic p27 re-expression only partially rescued apoptosis in p27 knockdown cells, it is likely that both p27 functions (apoptosis suppression in the cytoplasm and CDK2 inhibition in the nucleus) play roles in modulating apoptotic responses to lapatinib treatment

Although the mechanisms by which cytoplasmic p27 blocks apoptosis are unknown, this appears to be a widespread feature of cancer cells, and related findings have been reported in mitogen-depleted MCF7 breast cancer cells [12] and in U87-MG glioma cells, that express abundant cytoplasmic p27. One proposed mechanism involves sequestration of the tumor suppressor PTEN by cytoplasmic p27, which inhibits PTEN by preventing its nuclear translocation [33]. AKT is thought to prevent p27 nuclear localization in breast cancer cells by phosphorylating p27 within its nuclear localization signal on T157 [20, 25, 26]. Moreover, in renal cell carcinoma cells, inhibition of the PI3K/AKT pathway reduced p27 T157 phosphorylation and restored its nuclear localization [34]. Since Her2 signaling activates AKT, we considered that lapatinib might promote p27 nuclear relocalization by inhibiting phosphorylation of p27 by AKT. However, whereas lapatinib treatment decreased AKT activity in Her2+ cells, we found that p27 cytoplasmic localization was unaffected by inhibition of AKT activity using an AKT specific inhibitor (Supplemental Figs. 2A–2B). Thus, other pathways(s) down stream of Her2 exist which controls the cytoplasmic retention of p27. Identification of such pathway(s) would not only improve our understanding of the mechanism of p27 mislocalization but also provide insight into new therapeutic targets.

## MATERIALS AND METHODS

### Cell culture and viral infection

All cell lines were purchased from the American Type Culture Collection (ATCC). The following Her2− human breast cancer cell lines were used: HCC38, HCC1395, HCC70, MDA231, MDA468, HCC1806, HCC1143 and HCC1937. The following HER2+ breast cancer cell lines were used: HCC202, HCC1569, HCC1419, BT474, UACC893, CRL2351 and HCC1954. UACC893, MDA468 and BT474 cell lines were cultured in Dulbecco's modified Eagle's medium (DMEM) (Invitrogen) supplemented with 10% fetal bovine serum (FBS) (Hyclone), and 1% penicillin/streptomycin (P/S). All other cancer cell lines (HCC38, HCC1937, HCC1395, HCC70, HCC1806, HCC1143, HCC202, HCC1569, HCC1419, CRL2351, HCC1954 and MDA231) were cultured in Roswell Park Memorial Institute (RPMI) 1640 medium (Invitrogen), supplemented with 10% FBS and 1% P/S. MCF10A cells were grown in DMEM/F12 medium supplemented with 5% horse serum, 10 μg/ml insulin, 20 ng/ml EGF, 0.5 μg/ml hydrocortisone, 100 ng/ml Cholera toxin and 1% P/S. Cells were grown at 37°C in 5% CO2. Retroviral and lentiviral infections were performed as previously described [35].

### Plasmids, antibodies and other reagents

Lentiviral vector GIPZ-p27shRNA (V3LHS_410220) targeting the 3′-UTR of p27 was purchased from Thermo Scientific. The shorthair pin sequences are as follows:

TGCTGTTGACAGTGAGCGCCACAATAACACT AAAATTTTATAGTGAAGCCACAGATGTATAAAATT TTAGTGTTATTGTGTTGCCTACTGCCTCGGA. P27 mutants were generated by site-directed mutagenesis and full length p27 mutants were subcloned into the retroviral pBABE-hyrgomycin.

Primary antibodies used for western blots were: p27 (sc-528; Santa Cruz Biotechnology), γ-tubulin (T6557; Sigma-Aldrich), Flag (F3165; Sigma-Aldrich), Akt (Cat#9272, Cell Signaling), phospho-Akt (Ser473) (Cat#9271, Cell Signaling). For flow cytometry, BrdU antibody (Cat#556028; BD Pharmingen) was used. For immunostaining, the primary antibodies included BrdU antibody (ab8152; Abcam), cleaved caspase 3 (Cat#9664, Cell Signaling), and p27 (119–2; generated in the lab). Alexa Fluor-488 or Alexa Fluor-647 conjugated goat anti-rabbit or goat anti-mouse (Invitrogen) were used as secondary antibodies for immunostaining. Lapatinib is from BioVision (Cat#1624–100).

### Western blotting

Cells were lysed in buffer consisting of 50 mM NaCl, 1 mM EDTA, 2.5 mM EGTA, 0.1% Tween-20, 10% glycerol, 1% Nonidet P-40, and a protease/phosphatase inhibitor cocktail. 30 μg of protein was loaded onto SDS-PAGE gels for western blotting. For detection of p27, γ-tubulin and Akt, membranes were blocked in buffer consisting of PBS, 0.1% Tween-20 and 5% milk for 10 min at room temperature. For detection of phosphorylated Akt, membranes were blocked in buffer consisting of TBS, 0.1% Tween-20 and 5% BSA for 1 hr at room temperature. Membranes were then incubated with primary antibodies overnight at 4°C, followed by incubation with secondary antibodies for 2 hrs at room temperature.

### Immunofluorescence and quantitation

Cells were seeded on glass coverslips and treated as described, followed by fixation in 2% paraformaldehyde in PBS at 37°C for 20 min. For detection of p27 and caspase 3, cells were permeabilized for 6 min in 0.2% Triton-X100, blocked for 30 min in blocking buffer (1x PBS, 0.1% goat serum) and stained for 1.5 hrs with primary antibodies. The coverslips were then washed and incubated with Alexa Fluor-647-conjugated secondary antibody for 30 min in the dark. After washing, coverslips were mounted on glass slides with ProLong Gold Antifade reagent containing DAPI (Invitrogen). BrdU immunostaining was performed according to the manufacture's protocol (Abcam) with some modification. Briefly, for p27 and BrdU double immunostaining, cells were pulse labeled with 25 μM of BrdU for 5–13 hrs before fixation. After p27 immunostaining and washing, coverslips were incubated in HCl (2N) for 25 min at room temperature followed by incubation for 20 min at 37°C. Coverslips were washed and incubated in PBS containing 0.1 M of Na-Borate for 12 min at room temperature to neutralize HCl. Coverslips were then washed and incubated with anti-BrdU antibody for 1 hour. After washing, coverslips were incubated with Alexa-488 conjugated secondary antibody for 30 min in dark. Coverslips were washed and mounted on glass slides with anti-fade containing dapi. Slides were visualized on a Nikon Eclipse 800 microscope and images were obtained with a 20 × objective using a Spot charge-coupled-device camera (Diagnostic Instrument).

For quantification of p27 localization, cells from at least 50 cell clusters were counted. For each cluster, if the ratio of cells with p27 exclusively in the nucleus to cells with p27 exclusively in the cytoplasm was greater than 1, the cell cluster was scored as “nuclear” (n). If the ratio is less than 1, the cell cluster was scored as “cytoplasmic” (c). If the ratio was equal to 1, the cell cluster was scored as “cytoplasmic + nuclear” (n + c). For quantification of apoptotic cells, caspase 3 positive cells were counted from at least 10 cell clusters.

### Cell proliferation and drug sensitivity assay

For analysis of cell proliferation, cells were seeded at a density of 3 × 10^5^ cells/60mm plate. At the indicated time points, cells were trypsinized and viability was determined by trypan blue exclusion. Cells were counted using a hemocytometer. For drug sensitivity assays, cells were incubated in serum free medium for 72 hrs for synchronization 2 days after seeding. Cells were then switched into medium with serum for 48 hrs followed by lapatinib treatment for 72 hrs. Cell viability was determined by trypan blue exclusion. The survival rate was calculated by normalizing the cell number under drug treatment to vehicle (DMSO treatment). Experiments were repeated for three times.

### Flow cytometry and cell cycle analysis

Cells were pulse labeled with BrdU (100 mM) for 2 hrs before harvest. Cells were fixed and stained with a BrdU antibody and Propidium idoide, as previously described [36]. Cell cycle distribution was analyzed using CELLQUEST software.

## SUPPLEMENTARY FIGURES


