# Supplementary material for: Mislocalization of p27 to the cytoplasm of breast cancer cells confers resistance to anti-HER2 targeted therapy
Source: Oncotarget. 2015 Jan 3;5(24):12704–14. doi: 10.18632/oncotarget.2871 (PMC4350358; doi:10.18632/oncotarget.2871)
Supplement: Supplementary file 1 [file oncotarget-05-12704-s001.pdf]

SUPPLEMENTARY FIGURES

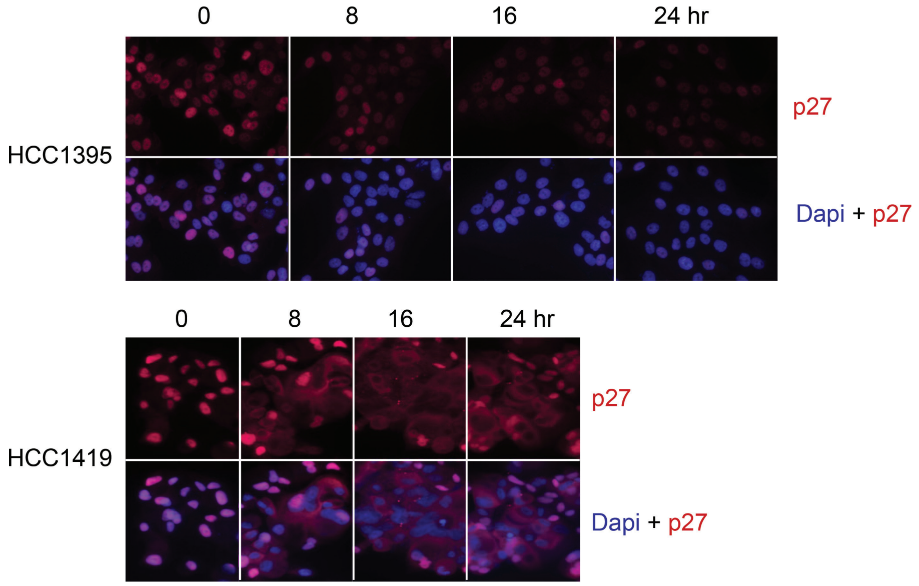

Supplementary Figure 1: P27 mis-localizes to the cytoplasm in HER2+ breast cancer cells. Immunofluorescence staining of p27 from HCC1395 (HER2−) and HCC1419 (HER2+) cells harvested at the indicated time points after released from serum starvation.

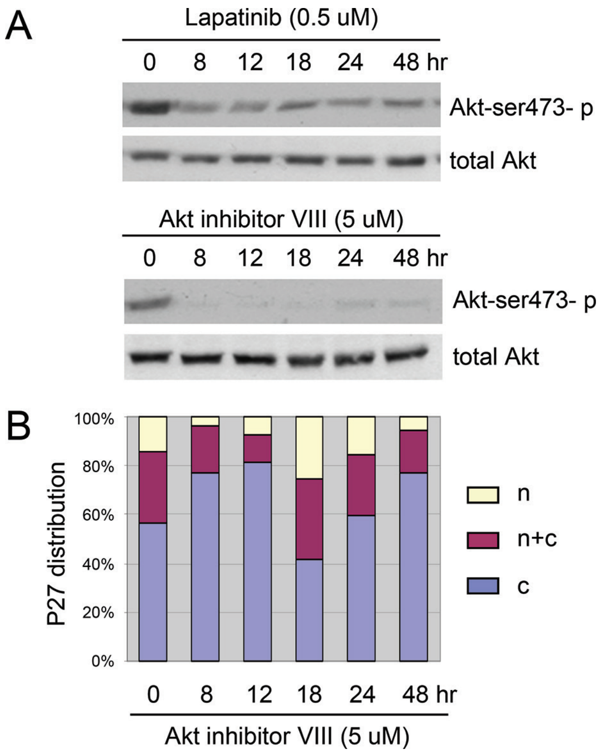

Supplementary Figure 2: Akt inhibition did not induce p27 nuclear relocalization in HER2+ cells. UACC893 cells were released from serum starvation for 48 hr. and treated with lapatinib or Akt inhibitor Viii as indicated. Western blots of total Akt and Akt phosphorylated at Ser 473 **(A)** as well as p27 immunostaining **(B)** are shown. Subcellular distribution of p27 was quantified as in Fig. 3A.
